# A Case of Streptococcus anginosus Brain Abscess Caused by Contiguous Spread from Sinusitis in an Immunocompetent Patient

**DOI:** 10.7759/cureus.1745

**Published:** 2017-10-04

**Authors:** Nathan Esplin, John W Stelzer, Sean All, Sundeep Kumar, Ejaz Ghaffar, Sayed Ali

**Affiliations:** 1 University of Central Florida College of Medicine; 2 Department of Internal Medicine, Osceola Regional Medical Center; 3 Medicine, Orlando VAMC

**Keywords:** streptococcus anginosus brain abscess, brain abscess, streptococcal infections, neuroimaging, mri brain, ct brain

## Abstract

Brain abscesses are infections of the brain parenchyma that can arise from either contiguous spread from local infection or by hematogenous spread from a distant site. Streptococcus anginosus of the Streptococcus anginosus group (SAG) is a commensal microbe of the mucosae of the oral cavity, gastrointestinal tract, and urogenital tract. We present a case of mono-microbial brain abscess caused by contiguous spread from relatively asymptomatic sinusitis that initially presented as a subdural hemorrhage on computed tomography.

A 70-year-old male presented, obtunded, with a Glasgow Coma Score of eight. The patient seized on arrival. A computed tomography scan was read as a subdural hemorrhage, and magnetic resonance imaging showed a heterogeneous area at the anterior tip of the left frontal lobe interpreted as a frontoparietal abscess, along with pansinusitis. Craniotomy revealed a loculated abscess. Culture grew only Streptococcus anginosus. The patient did well postoperatively, was extubated by day five with rapidly improving neurological function, and was discharged to inpatient rehab by hospital-day eight for the continuation of intravenous antibiotics.

This case represents a frontal lobe abscess caused by the contiguous spread of Streptococcus anginosus from a frontal sinus infection. This is a relatively rare presentation of SAG infection in an immunocompetent patient. The case outlines the importance of imaging modality choice in the various stages of brain abscess formation, and the necessity of maintaining an index of suspicion for brain abscess in patients with few traditional risk factors and little to no history on presentation.

## Introduction

Brain abscesses are infections of the brain parenchyma that can arise from either the contiguous spread of local infection or by the hematogenous spread from a distant site. Streptococcus anginosus is a commensal microbe of the mucosae of the oral cavity, gastrointestinal tract, and urogenital tract [1]. We present a case of, apparently, mono-microbial brain abscess in an immunocompetent individual believed to be caused by the contiguous spread of Streptococcus anginosus from a sinus infection that initially mimicked a subdural hematoma on computed tomography (CT).

## Case presentation

The patient is a 70-year-old male who was found in a chair at home by emergency medical services after a call from a friend who did not have his usual contact with the patient for several days. When paramedics arrived, it appeared he had been in that position for some time, as he had been incontinent of both bowel and bladder. He was found to be non-verbal, responsive only to painful stimuli, and was able to move all extremities, giving him a Glasgow Coma Scale (GCS) of eight. Medical history obtained from the friend who called emergency services was largely incomplete but was positive for diabetes mellitus. The patient had no reported history of stroke. Upon arrival to the emergency department, the patient began to seize with right gaze deviation. Lorazepam was administered to control the seizure and an emergent intubation was performed to protect his airway. The patient was afebrile, and vital signs were within normal limits throughout the course of presentation and treatment. On physical exam, there was a mild left facial droop with 3 mm pupils that were equal and reactive to light. The rest of his exam was non-focal. Laboratory results demonstrated a leukocytosis of 20,720 per cubic millimeter of blood and mild electrolyte disturbances.

**Figure 1:**
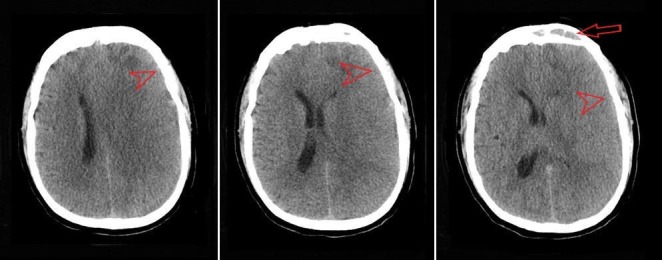
Non-contrast computed tomography (CT) showing possible subacute left subdural hematoma (arrowhead) and suggesting frontal sinusitis (arrow)

**Figure 2:**
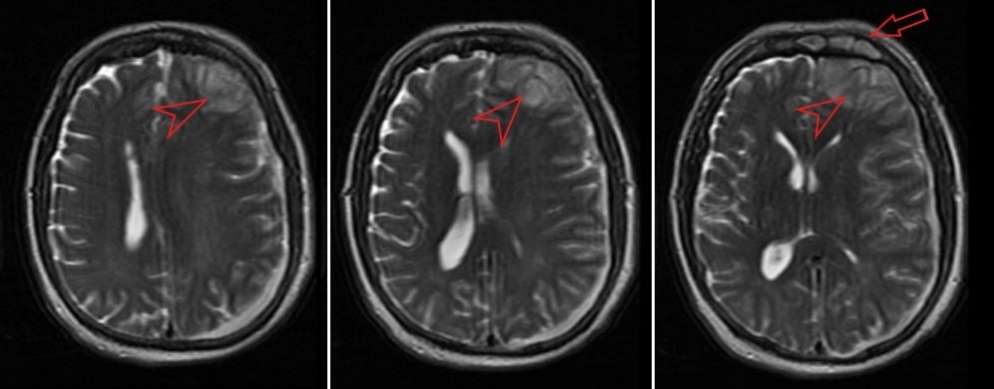
T2 weighted magnetic resonance imaging (MRI) showing complex left-sided acute subdural fluid collection with left frontal abscess (arrowhead), and sinusitis (arrow).

A computed tomography (CT) scan performed in the emergency department showed a crescent-shaped hemorrhage with midline shift consistent with a subdural hemorrhage (Figure 1). The CT was read by the radiologist as, “Subacute left subdural hematoma, 1.4 cm in width with midline shift from left to right of 0.7 cm. Left frontal encephalomalacia versus involving Q skin nick event.” The most likely differential diagnosis now included subdural hematoma vs. empyema. It was felt that the symptomatology did not overlap completely with subdural hemorrhage so magnetic resonance imaging (MRI) was performed. It showed a complex left-sided acute subdural fluid collection of moderate size with mass effect upon the left cerebral hemisphere and a 6.3 mm midline shift to the right (Figure 2). It also showed a heterogeneous area at the frontal tip of the left frontal lobe that was interpreted as a frontoparietal abscess. The MRI also revealed enhancement of the frontal sinuses consistent with frontal sinusitis. History taken from family members days later was positive for mild, self-treated, sinusitis-like symptoms immediately prior to his presentation.

An emergent frontoparietal craniotomy was performed, and a loculated abscess was found in the anterior tip of the left frontal lobe. It was evacuated and the space was irrigated with bacitracin. Prior to irrigation, samples were taken from the subdural space and the abscess.

The patient did well postoperatively and by postoperative day five he was extubated and verbal, but with some anomic aphasia. Hemiparesis was present immediately postoperative but improved rapidly along with his facial droop. Cultures from the abscess grew Streptococcus anginosus. He did have some seizure activity, myokymia of the face, and left upper extremity tremors on postoperative day one, which were controlled with levetiracetam 1000 mg, twice per day. The patient continued to improve neurologically and was well enough to be discharged on postoperative day eight to an inpatient rehab facility for rehabilitation and continued intravenous antibiotics.

## Discussion

Brain abscesses are infections of the brain parenchyma that can arise from both contiguous spread through emissary veins, or by hematogenous spread from a distant site [2]. Generally, brain parenchyma is resistant to infection. Patients who present with brain abscesses usually have predisposing factors such as immunosuppression, penetrating trauma, neurosurgical procedures, or a focus of infection in the sinuses, or one that leads to recurrent bacteremias like endocarditis or arteriovenous malformation. Hematogenous cases tend to be multiple, arise in the middle cerebral artery territory, and are often located in the grey-white matter interface [3]. Abscesses from direct spread are usually singular and are found adjacent to their source- for example, infections spreading from a sinus infection will be in the frontal lobes [2]. Brain abscesses tend to progress through a pattern of early cerebritis progressing to encapsulation. The encapsulation process involves neovascularization, fibroblast accumulation, and gliosis, often leading to seizures during the encapsulation phase and as long-term sequelae [4]. The microbiology of brain abscess depends on the immune status of the patient, other foci of infection, and history of trauma or instrumentation [2].

In the last several years, we are beginning to understand that Streptococcus anginosus is a more important pathogen than previously thought. It is a member of the viridans group Streptococci. It is very similar to Streptococcus constellatus and Streptococcus intermedius. The three together are often called the Streptococcus anginosus group (SAG), but were previously and occasionally still referred to as Streptococcus milleri group. Streptococcus (S.) anginosus is a commensal microbe of the mucosae of the oral cavity, gastrointestinal tract, and urogenital tract. It is unique in the viridans group for forming abscesses and causing endocarditis. It is encapsulated, and it has poorly studied and incompletely understood properties that allow it to adhere well to human tissues [1, 5]. Infection with SAG in pediatric rhinosinusitis is being recognized as tending to have a worse prognosis or a tendency to more serious complications as compared to other pathogens [6]. The literature contains several case reports of disseminated infection with Streptococcus anginosus group bacteria, but in those cases, there are often multiple abscesses and represent the hematogenous spread of the bacteria. In one review, the brain was involved in over 90% of cases of disseminated SAG infections, with additional sites of infection in roughly 50% of cases [7].

Treatment of brain abscess with SAG infection nearly always requires neurosurgical intervention [2]. Compared to other pathogens, it often requires more aggressive surgical intervention and longer courses of antibiotic treatment [6]. Beta-lactam antibiotics are often effective, with ceftriaxone being a popular choice in treatment [8]. Laboratories may not always report local susceptibilities, so an attempt should be made to find current susceptibility information in the literature and to attempt to trend susceptibilities in local communities.

This case presented a singular, loculated abscess in the anterior tip of the left frontal lobe of a patient who was relatively immunocompetent. It appeared to have ruptured, with the infection spreading locally to result in an empyema that mimicked the appearance of a subdural hematoma on the initial CT scan. Aside from the location of the abscess and the fact that it was a solitary lesion, the MRI demonstrated enhancement in the frontal sinus, most likely signifying infection, providing evidence to suggest local spread from the sinus to the frontal lobe.

The unique aspect of this case is the unusual presentation of a contiguous abscess formation in an immunocompetent patient. It is also worth noting that it initially mimicked a subdural hematoma. The undefined initial CT complicated the patient’s initial workup. The abscess could not be confirmed until magnetic resonance imaging was obtained. The correct imaging modality is crucial to determine the specific pathology affecting the patient, but it must also be correlated to relevant lab work and more importantly the patient’s history and clinical picture. It is well established that subdural or parenchymal empyemas are most reliably diagnosed by MRI. Additionally, CT is often non-specific regarding the substance of the lesion and its intracranial location. Further differentiation between empyemas and sterile effusions or hematomas is performed in comparing the suspected fluid with cerebrospinal fluid and white matter on both T1 weighted MRI and T2 weighted MRI [9]. With this information, one could argue that a patient without a history of head trauma should receive an emergent MRI for suspicion of abscess formation or empyema. However, the case is further misleading because an elderly man who is apparently immunocompetent except for the inherent immunocompromise of age, and without a reported history of recent or previous infection would not necessarily point toward an infectious intracranial process. Lab work such as C-reactive protein (CRP) and erythrocyte sedimentation rate (ESR) can help to further delineate differences in the imaging findings, as they have been used in previous studies to not only identify the presence of infection, but also guide the management of brain abscess resolution altogether.

## Conclusions

*​​​​​​​*This case represents a frontal lobe abscess caused by contiguous spread of Streptococcus anginosus from a frontal sinus infection. The case outlines the importance of imaging modality choice in the various stages of brain abscess formation. Imaging must always be correlated with the patient’s presentation, history, and associated risk factors for disease. It also demonstrates that it is important to maintain an index of suspicion for brain abscess in patients with few traditional risk factors and little to no history on presentation.
